# Depletion of branched‐chain aminotransferase 2 (BCAT2) enzyme impairs myoblast survival and myotube formation

**DOI:** 10.14814/phy2.14299

**Published:** 2019-12-12

**Authors:** Zameer N. Dhanani, Gagandeep Mann, Olasunkanmi A. J. Adegoke

**Affiliations:** ^1^ School of Kinesiology and Health Science Muscle Health Research Centre York University Toronto ON Canada

**Keywords:** BCAT2, branched‐chain amino acids, differentiation, metabolism, myotube

## Abstract

Much is known about the positive effects of branched‐chain amino acids (BCAA) in regulating muscle protein metabolism. Comparatively much less is known about the effects of these amino acids and their metabolites in regulating myotube formation. Using cultured myoblasts, we showed that although leucine is required for myotube formation, this requirement is easily met by α‐ketoisocaproic acid, the ketoacid of leucine. We then demonstrated increases in the expression of the first two enzymes in the catabolism of the three BCAA, branched‐chain amino transferase (BCAT2) and branched‐chain α‐ketoacid dehydrogenase (BCKD), with ~3× increase in BCKD protein expression (*p* < .05) during differentiation. Furthermore, depletion of BCAT2 abolished myoblast differentiation, as indicated by reduction in the levels of myosin heavy chain‐1, troponin and myogenin. Supplementation of incubation medium with branched‐chain α‐ketoacids or related metabolites derivable from BCAT2 functions did not rescue the defects. However, co‐depletion of BCKD kinase partially rescued the defects. Collectively, our data indicate a requirement for BCAA catabolism during myotube formation and that this requirement for BCAT2 likely goes beyond the need for this enzyme to generate the α‐ketoacids of the BCAA.

## Graphical abstract

Although branched‐chain amino acids (BCAA) and their metabolites can regulate muscle mass by stimulating muscle protein synthesis, very little is known about their roles in regulating muscle cell (myotube/myofiber) formation. Our data demonstrate that BCAT2, the enzyme that catalyzes the first step in BCAA catabolism, is required for myotube formation. This requirement cannot be met by providing the ketoacids of the BCAA; rather, activating branched‐chain α‐ketoacid dehydrogenase complex activity partially rescues the defects. Thus, in addition to regulating protein synthesis, proper regulation of BCAA metabolism is vital for myotube formation.

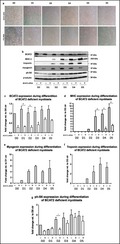

## 1 INTRODUCTION

Branched‐chain amino acids (BCAA: leucine, isoleucine, and valine) alone or in combination with resistance exercise have anabolic effects on skeletal muscle. Consistent with this, BCAAs, and in particular leucine, can induce signaling that promotes muscle protein synthesis via the mammalian/mechanistic target of rapamycin complex 1 (mTORC1) (Anthony, Anthony, & Layman, 1999; Matsumoto et al., 2007), inhibit protein degradation via the ubiquitin‐proteasome system (Herningtyas et al., 2008), improve mitochondrial metabolism (D’Antona et al., 2010), and improve glucose transport and insulin signaling in some (Kleinert et al., 2011; Liu et al., 2014; Nishitani et al., 2002) though not all (Moghei, Tavajohi‐Fini, Beatty, & Adegoke, 2016) studies. Interestingly, many of the anabolic features of BCAA can be mimicked by metabolites of these amino acids. For example, the ketoacid of leucine, α‐ketoisocaproic acid (KIC), can activate mTORC1 and stimulate muscle protein synthesis (Escobar et al., 2010; Lynch et al., 2003; Moghei et al., 2016; Xu, Kwon, Cruz, Marshall, & McDaniel, 2001).

The first step in the catabolism of BCAA is the reversible conversion of these amino acids to their corresponding α‐ketoacids (α‐ketoisocaproate (KIC), α‐keto‐β‐methylvalerate (KMV), and α‐ketoisovalerate (KIV), respectively from leucine, isoleucine and valine). Because α‐ketoglutarate is the usual amino group acceptor in the transamination reactions of the BCAA, glutamate is also produced. In skeletal muscle, the formation of the ketoacids from the three BCAA is catalyzed by a single enzyme, the mitochondrial branched‐chain aminotransferase 2 (BCAT2 or BCATm). The ketoacids can then be irreversibly oxidatively decarboxylated to their acyl‐CoA derivatives (isovaleryl‐CoA, 2‐methylbutryl‐CoA and isobutyryl‐CoA, respectively from KIC, KMV and KIV) by the branched‐chain ketoacid dehydrogenase complex (BCKD). The resulting acyl‐CoA derivatives can either be oxidized to yield ATP, CO_2_ and H_2_O, or enter alternative pathways. One of such pathways is the utilization of leucine‐derived β‐hydroxyl‐ β‐methylglutaryl‐CoA (HMG‐CoA) for de novo cholesterol synthesis, an essential component in membrane biogenesis (Berg, Tymoczko, & Stryer, 2002). Unlike the metabolism of the other BCAA, KIC can also be used to make β‐hydroxy‐β‐methylbutyrate (HMB), a compound with protein anabolic effect in skeletal muscle (Girón et al., 2016).

Alterations in BCAA metabolism and metabolites affect whole body and skeletal muscle metabolism. For example, BCAT2 knock out mice have increased body weight, augmented protein turnover and whole body insulin sensitivity but without an effect on skeletal muscle weight (She et al., 2007). However, these animals, which have increased circulating levels of BCAA but lower levels of the corresponding ketoacids and of glutamine, display impaired exercise capacity (She et al., 2010). Defects in BCKD activity, such as are seen in individuals with maple syrup urine disease, are associated with profound muscle and movement abnormalities (Carecchio et al., 2011; Ferrière, Castro, & Rodriguez, 1984; Friedrich, Lambert, Masino, & Downes, 2012; Jouvet, Kozma, & Mehmet, 2000; Jouvet et al., 1998). Finally, mice with muscle specific deletion of BCKD kinase (BDK, the enzyme that catalyzes the inhibitory phosphorylation of the E1α subunit of BCKD), which would lead to elevated BCKD activity, have exaggerated muscle catabolic response to protein deficiency (Ishikawa et al., 2017). These studies underline the significance of appropriate regulation of muscle and whole body BCAA catabolism in organismic metabolism and response to stressors.

Although the significance of BCAA in muscle growth and metabolism is incontrovertible, comparatively much less is known on the roles of these amino acids and their metabolites during muscle cell differentiation. Muscle cell differentiation is a vital process in converting mono‐nucleated proliferating myoblasts to terminally differentiated, multi‐nucleated myotubes, the latter being the building blocks for forming new myofibers or repairing existing ones. When myotubes are formed, there is a change in the proteome such that there is an abundance of myofibrillar proteins, including myosin heavy chain, troponin, and tropomyosin. Since these new proteins need to be synthesized de novo, and given the significance of BCAA in regulating protein turnover, it is reasonable to envisage a role for these amino acids during differentiation. Moreover, there is a requirement for mTORC1 activation during differentiation (Erbay & Chen, 2001). BCAA might facilitate such an activation. Muscle cells can use leucine as a source of cholesterol (Miettinen & Penttilä, 1968), which potentially can be used in making myotube membranes during differentiation. Leucine can also serve as a fuel for oxidative metabolism (Bowtell, Marwood, Bruce, Constantin‐Teodosiu, & Greenhaff, 2007) during differentiation, a process that is associated with increased mitochondrial content and oxidative metabolism (Malinska, Kudin, Bejtka, & Kunz, 2012). Along this line, myoblasts cultured in leucine‐free medium do not differentiate well, a defect that is associated with abnormal regulation of myogenic regulatory factors (MRFs) myf5 and myoD (Averous, Gabillard, Seiliez, & Dardevet, 2012; Dai et al., 2015). In addition, HMB positively regulates the differentiation of human and chicken skeletal muscle myoblasts (Kornasio et al., 2009). These studies suggest a role for BCAA and their metabolites in regulating muscle cell differentiation.

Here, we first examined the effects of leucine on myoblast differentiation. Our data revealed that while leucine is required for differentiation of L6 myoblasts, this requirement can be met by KIC supplementation. In addition to reporting an increase in the levels of the first two enzymes in BCAA catabolic pathway during differentiation, we demonstrated a requirement for BCAT2 for optimal myotube formation and showed that this requirement likely goes beyond its ability to produce the α‐ketoacids from the BCAA.

## 2 MATERIALS AND METHODS

### 2.1 Reagents

Fetal Bovine Serum (FBS), Horse Serum (HS), Lipofectamine RNAiMAX, OptiMEM, and antibiotic/antimycotic reagents were purchased from Life Technologies. Pierce BCA Protein Assay Kit and RNA isolation kits were obtained from Thermo Fisher; leucine‐free medium (RPMI 1640) from US Biologicals; alpha ketoisocaproic acid (KIC), phosphatase and protease inhibitor cocktails, scramble, BDK and BCAT2 siRNA oligonucleotides were purchased from Sigma‐Aldrich. α‐Modification of Eagle's Medium (AMEM) and Phosphate Buffered Saline (PBS) were obtained from Wisent.

### 2.2 Antibodies

Antibodies to branched‐chain α‐ketoacid‐dehydrogenase E1 α‐polypeptide (BCKDE1α), BCAT2 and to γ‐tubulin were purchased from Sigma–Aldrich. Antibodies to phosphorylated ribosomal protein S6 (S235/236), phosphorylated AKT (S473), caspase 3, caspase 7, poly (ADP‐ribose) polymerase (PARP), GAPDH, and HRP‐conjugated rabbit and mouse IgG were purchased from Cell Signaling Technology. Antibodies to troponin, myogenin and myosin heavy chain‐1 (MHC‐1) were obtained from Developmental Studies Hybridoma Bank.

### 2.3 Cell culture and differentiation

L6 rat myoblasts were obtained from the American Type Culture Collection. Cells (5.0 × 10^5^) were seeded in 10‐cm plates in growth medium (GM) (AMEM supplemented with 10% fetal bovine serum and 1% antibiotic‐antimycotic agents) and incubated at 37°C and 5% CO_2_. To initiate differentiation, cells were first grown to ~90% confluency in 6‐well plates and then shifted into differentiation medium (DM) consisting of AMEM, 1% antibiotic‐antimycotic agents, and 2% horse serum. DM was replaced every 24 hr.

### 2.4 Leucine deprivation and KIC rescue experiments

To examine how differentiation was affected by depriving myoblasts of leucine, 1.5 × 10^5^ cells were cultured in 6‐well plates. Once cells reached ~90% confluency, the medium was changed to the differentiation medium consisting of RPMI 1640, 2% horse serum, and 1% antibiotic‐antimycotic agents. This RPMI medium was used because it lacks l‐leucine, l‐glutamine, and sodium bicarbonate). l‐glutamine and sodium bicarbonate were re‐added to all RPMI media at concentrations to mimic their levels in common growth media. RPMI differentiation media was changed every 24 hr. To test if the leucine metabolite KIC can rescue leucine‐deprived cells, cells were differentiated in a medium that was leucine‐free, or leucine‐free but supplemented with 200 µM KIC. The supplemented differentiation medium was changed every 24 hr. On D0 to D5 of differentiation, batches of cells were washed with cold PBS and harvested with 100 µl lysis buffer (1 mM EDTA, 2% sodium dodecyl sulfate (SDS), 25 mM Tris, 1 mM DTT, supplemented (10 µl/ml) with each of protease and phosphatase inhibitor cocktails. Samples were stored at −20°C.

### 2.5 Effect of BCAT2 depletion on myoblast differentiation

We utilized RNAi to deplete myoblasts of BCAT2, using lipofectamine RNAiMAX (Life Technologies). Briefly, 2.5 × 10^5^ cells were seeded in 6‐well plates along with either scramble (control, Sigma #SIC001) or BCAT2 siRNA oligonucleotides (sense 5′‐CUAUGUGCGGCCGGUGCUU, anti‐sense 5′‐AAGCACCGGCCGCACAUAG) as described previously (Maeda, Abdullahi, Beatty, Dhanani, & Adegoke, 2017; Moghei et al., 2016). After 48 hr, cells were either harvested (D0 of differentiation) or washed twice with PBS and shifted into regular (complete) DM. At D1–D5, samples were harvested with 100 μl lysis buffer, and stored at −20°C for later analysis.

### 2.6 Rescuing differentiation defects in BCAT2‐depleted cells by increasing cell number

Because BCAT2 depletion reduced the number of viable cells (see Results section), we wondered if adding more cells could rescue the differentiation defects seen in BCAT2‐depleted myoblasts. To address this, 24 hr following transfection of cells with control or BCAT2 siRNA oligonucleotides in 6‐well plates, we trypsinized three wells of the BCAT2 siRNA‐treated cells and combined them into one new well. Similarly, for the control siRNA treated cells, we trypsizined one well and simply moved the cells into one new well. We only used one well for control cells because cell death was minimal in control cells compared to BCAT2 depleted cells, but those cells were trypsinized nevertheless to ensure identical handling between the two groups. Once the cells from both treatments were placed into new wells, they were allowed to grow in regular (complete) GM for another 24 hr. Cells were then shifted into regular DM and their ability to differentiate was examined.

### 2.7 Branched‐chain ketoacid (BCKA) rescue experiment in BCAT2 depleted cells

Because a primary function of BCAT2 is to generate the branched‐chain α‐ketoacids, we examined whether adding these ketoacids (KIC, KMV, and KIV) would rescue differentiation defects in BCAT2‐depleted cells. L6 myoblasts (2.5 × 10^5^) cells were transfected with control or BCAT2 siRNA transfection mix. Twenty‐four hours after transfection, 1 ml of regular GM was added to all wells. In BCAT2 siRNA‐treated wells, KIC, KIV, and KMV were also added to a final concentration of 200 μM for each of the ketoacids. After another 24 hr, the cells were shifted to regular DM, and again with or without added KIC, KIV, and KMV. DM was changed every subsequent 24 hr, with the stated ketoacids added as indicated above.

### 2.8 Effects of BCAT2 and BCKD kinase (BDK) co‐depletion on myoblast differentiation

To study the effect of BDK depletion on the differentiation of myoblasts with attenuated BCAT2 level, myoblasts were transfected with BCAT2 siRNA oligonucleotides as before, but with or without co‐transfection with BDK siRNA oligonucleotides (sense 5′‐CUAUGCAUGGCUUUGGCUU, anti‐sense 5′‐ AAGCCAAAGCCAUGCAUAG). Forty‐eight h later, myoblasts were harvested or cultured in regular DM. Cells were harvested at different times during differentiation and processed.

### 2.9 Western blotting

After determining protein concentrations in cell lysate, samples were run on either 10% or 15% SDS‐page gels and transferred to PVDF membranes. Western blot analysis was done as described previously (Maeda et al., 2017; Moghei et al., 2016). MHC‐1, BCKDE1α, and troponin were blotted for using the membranes from 10% gels, whereas, BCAT2, phosphorylated (ph) S6, caspase‐3, and myogenin were blotted for using the membranes from 15% gels.

### 2.10 mRNA analysis

Cells were differentiated as described above. RNA was isolated from sample harvested D0 to D5 using an RNA isolation kit (Thermo‐Fisher) and following manufacturer's instructions. RNA was stored at −80°C. cDNA was synthesized from RNA using a cDNA synthesis kit (Bio‐Rad). Sybr‐green chemistry based quantitative PCR (qPCR) was conducted using the Bio‐Rad SsoAdvanced™ Universal SYBR^®^ Green Supermix and analyzed using Bio‐Rad CFX96™ machine (Maeda et al., 2017). The following primers were used: BCAT2 [Forward: 5′‐TCCAGAACCTCACAGTGC‐3′ Reverse: 5′‐CCTGCTTGTCAAAGTCTG‐3′], BCKDE1α [Forward: 5′‐GGGCTTGGCTAGATTCA‐3’ Reverse: 5′‐GGGGATCTTCACTGGGGT‐3′], HPRT [Forward: 5′‐GCTTTCCTTGGTCAAGCAC‐3′ Reverse: 5′‐TCCAACAAAGTCTGGCCTGA‐3′]. We corrected for RNA quantity using the mRNA level of HPRT (hypoxanthine phosphoribosyltransferase 1).

### 2.11 Other analyses

Intracellular BCAA concentrations in cell lysate were measured as described previously (Beckett et al., 1996; Jeganathan, Abdullahi, Zargar, Maeda, & Riddell, 2014). We measured cell viability by using the CCK‐8 cell viability assay kit (Sigma #96992) and following the manufacturer's instructions. Cell viability is expressed relative to the cells cultured and differentiated in medium lacking siRNA reagents or siRNA oligonucleotides.

### 2.12 Statistical analysis

Two tailed non‐paired *t*‐test was used to assess the difference between two groups. One‐way ANOVA with Tukey Kramer post‐hoc test was conducted on experiments with more than two groups. Values are means ± *SEM*. Unless otherwise indicated, *n* = 3 independent experiments (biological replicates). Within each biological replicate, we assigned ≥wells per treatment. Statistical significant difference was determined as *p* < .05.

## 3 RESULTS

### 3.1 Leucine essentiality for differentiation can be met by KIC

**Figure 1:**
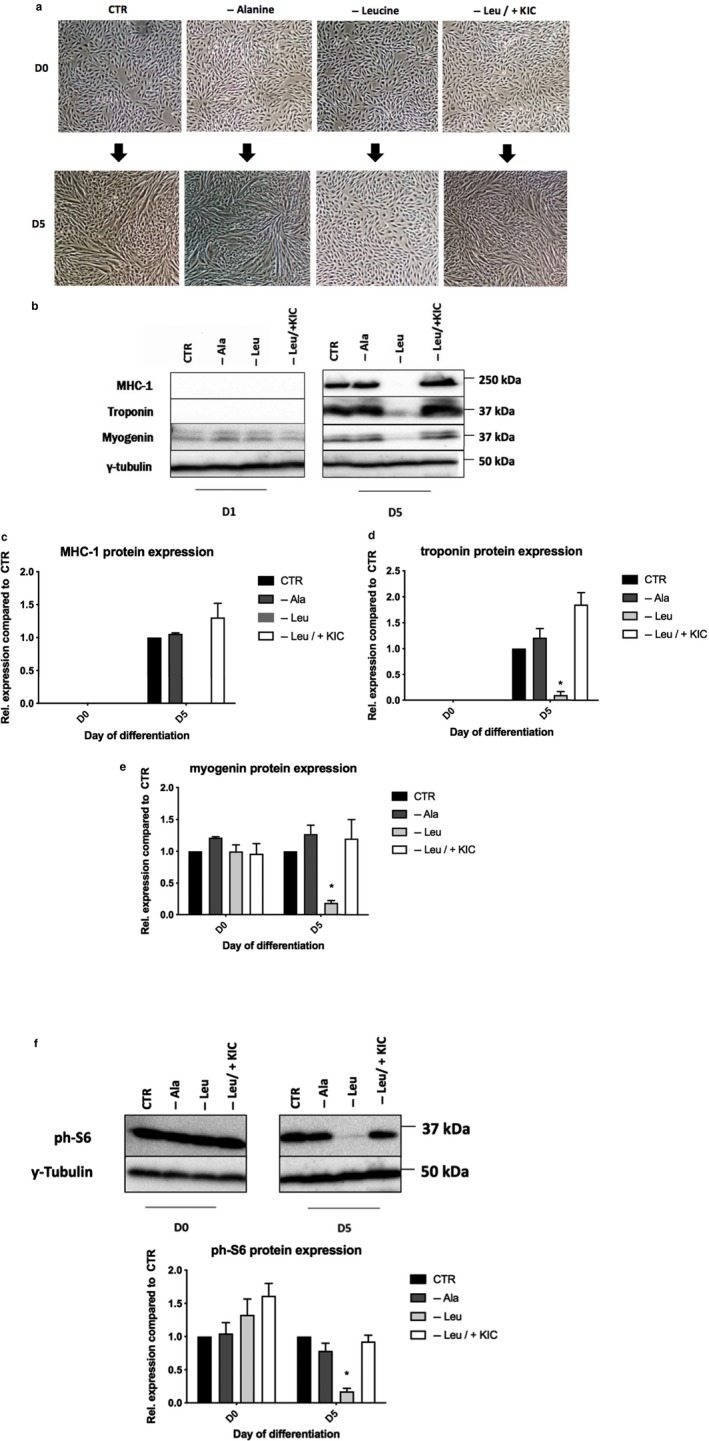
Leucine requirement during differentiation of myoblasts can be met by KIC. (a) L6 rat myoblasts were differentiated in either a differentiation medium (DM) with all amino acids (CTR), or DM lacking only l‐alanine, DM lacking only l‐leucine, or DM lacking only l‐leucine but also supplemented with 200 µM KIC for a period of 5 days. Day 0 (D0) represents the moment immediately before shifting the cells into the differentiation medium while day 5 (D5) represents the moment after allowing the cells to differentiate for 5 days. (b–e) Cells were harvested on day 0 (D0) and day 5 (D5) and probed for myosin heavy chain‐1 (MHC‐1), troponin, and myogenin, which are markers of differentiation*.* (f) Phosphorylated ribosomal protein S6 (a marker of mTORC1 signaling). In c–f, data are mean ± *SEM*; *n* = 3 independent experiments (i.e., biological replicates). *significantly different compared to all other conditions in the D5 group (*p* < .05)

Whereas myoblasts differentiated in the medium that lacked the non‐essential amino acid alanine, no differentiation was observed in cells grown in the absence of leucine, as indicated by the absence of myotubes (Figure 1a) and expression of myofibrillar proteins, including MHC‐1 and troponin and of myogenic regulatory factor myogenin (Figure 1b–e). The requirement for the essential amino acid leucine was fully met when its ketoacid, KIC, was provided. (Figure 1a–e). KIC also restored mTORC1/S6K1 signaling in cells lacking leucine, as reflected in the abundance of phosphorylated S6 ribosomal protein (Figure 1f). Conclusively, these results demonstrate the BCAA leucine is essential to L6 myoblast differentiation and that in its absence, KIC rescues the observed defects.

### 3.2 Expression of BCAA catabolic enzymes BCAT2 and BCKD increase during L6 differentiation

**Figure 2:**
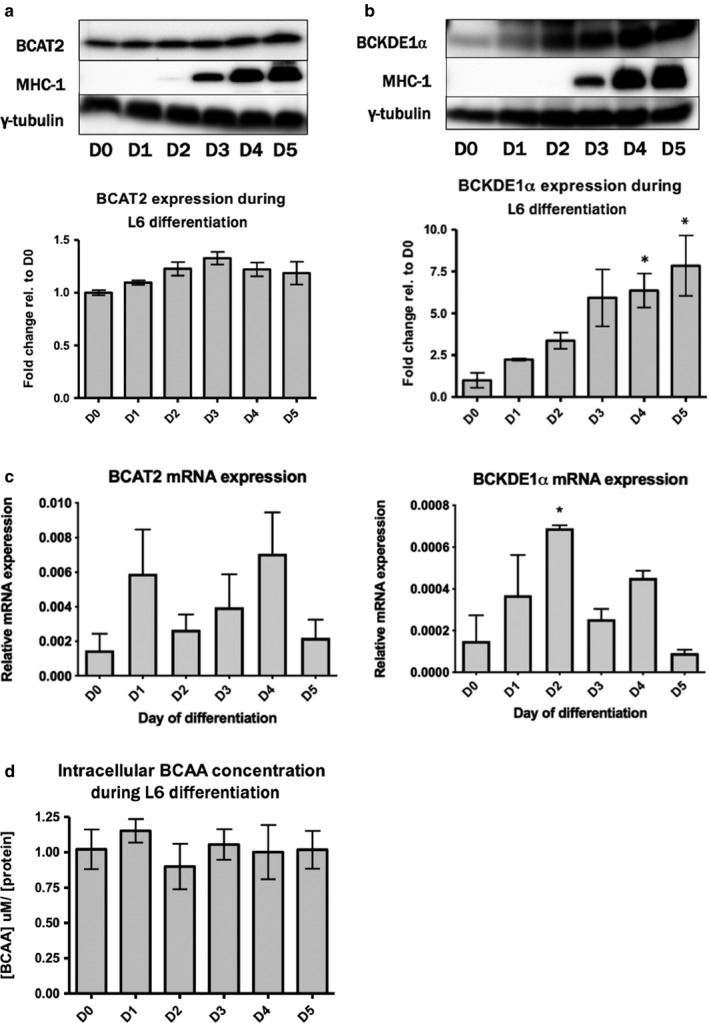
Expression of BCAT2 and BCKD during myoblast differentiation. (a) BCAT2 (branched‐chain aminotransferase‐2) and (b) BCKDE1α (E1α subunit of branched‐chain alpha‐ketoacid dehydrogenase) protein expression during differentiation of L6 myoblasts in regular DM. *significantly greater than day 1 (D1) (*p* < .05). (c) mRNA expression of BCAT2 and BCKDE1α during differentiation of L6 myoblasts. Data are mean ± *SEM*, *n* = 3 independent experiments. *significantly different from D5 (*p* < .05). (d) Intracellular BCAA concentration at each day of differentiation

Because we observed that KIC, a metabolite of leucine catabolism, could recapitulate the functions of leucine during differentiation, we wondered how the enzymes that catabolize the BCAA were regulated during myoblast differentiation. BCAT2 (the BCAT isoform found in skeletal muscle) was robustly expressed in myoblasts. Although the abundance of BCAT2 appeared highest on day 3 (D3) of differentiation, there was no statistically significant effect of differentiation on the abundance of this enzyme (Figure 2a). Expression of the E1α subunit of BCKD showed a significant trend to increase on all days of differentiation, where D4 and D5 were significantly higher compared to D1 of differentiation (*p* < .05) (Figure 2b). At the mRNA level, we found no statistically significant changes in BCAT2 expression. However, BCKDE1α mRNA was significantly higher on D2 of differentiation compared to D0, and thereafter declined (Figure 2c). The observed trends in BCAT2 and BCKD expression were not associated with any statistically significant change in intracellular levels of the BCAA over the 5‐day differentiation period (Figure 2d).

### 3.3 BCAT2 depletion prevents L6 myoblast differentiation

**Figure 3:**
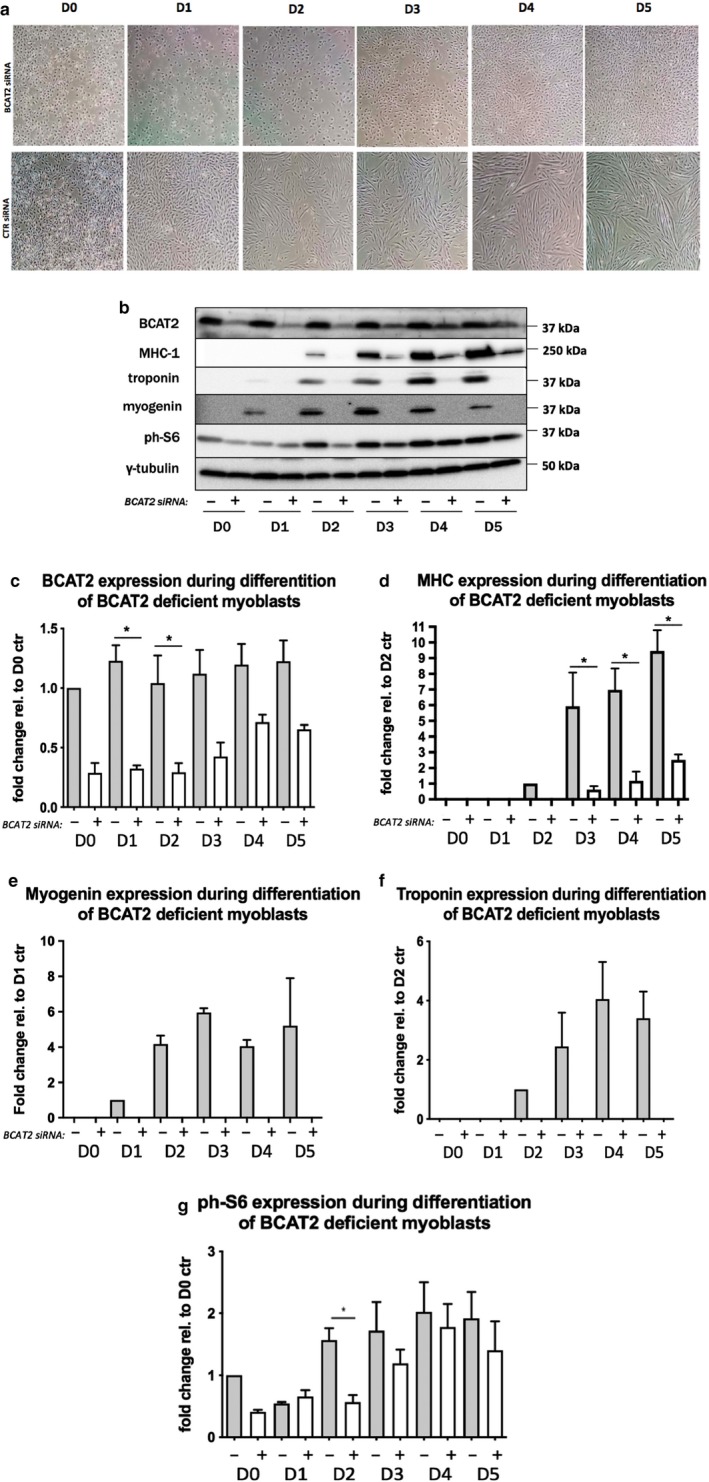
BCAT2 depletion impairs myotube formation. L6 rat myoblasts were transfected with control (CTR) or BCAT2 siRNA oligonucleotides. Two days later, myoblasts were harvested or shifted into regular DM. Samples were harvested on D1‐D5 of differentiation. (a) Light microscope images of cell during differentiation. Cells were harvested and probed for BCAT2 (b and c) and for myogenic proteins MHC‐1, troponin, and myogenin (b and d–f), and (g) Ribosomal protein S6 phosphorylation. Data are mean ± *SEM*; *n* = 3 independent experiments. *significant difference from corresponding scramble group (*p* < .05)

Our observation that KIC can meet the requirement for leucine and of increased expression of a critical subunit of BCKD during differentiation suggests a requirement for BCAT2 both for transamination of KIC back to leucine and to generate substrates for BCKD*.* Indeed, whereas cells transfected with scramble siRNA oligonucleotides (CTR siRNA) differentiated well, BCAT2 knockdown cells showed no visible myotubes (Figure 3a–c) and had markedly reduced levels of MHC (~20% of MHC in CTL siRNA group on D3) (Figure 3b and d). In addition, those cells had no detectable levels of troponin and myogenin (Figure 3b, e, and f). Interestingly, in cells depleted of BCAT2, phosphorylation of S6 was significantly reduced at D2 (*p* < .05) and showed a trend to be reduced at D3 and D4 as well (Figure 3b and g). Clearly, these results suggest that BCAT2 serves an essential role in the differentiation of myoblasts to myotubes.

### 3.4 Increasing cell confluency does not rescue differentiation defects in BCAT2‐depleted myoblasts

**Figure 4:**
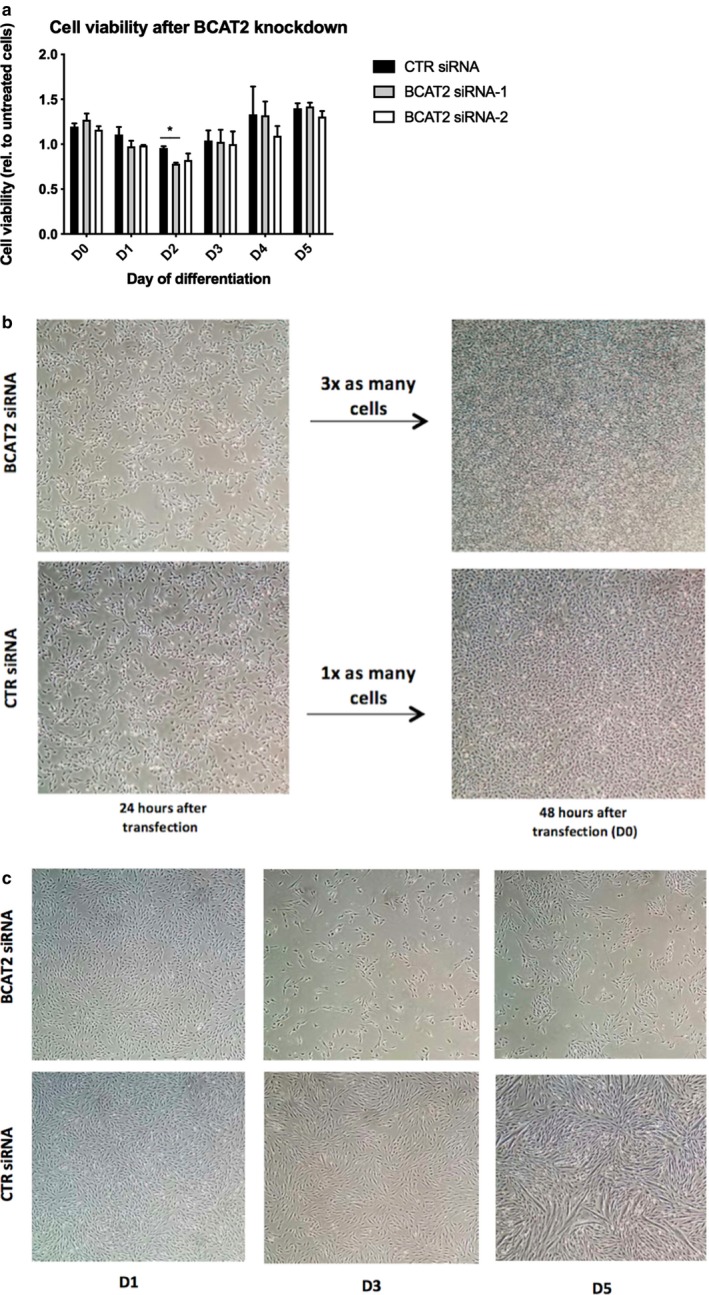
Differentiation defect in BCAt2‐depleted cells is not rescued by increasing cell confluency at the onset of differentiation. Cells were transfected with CTL or BCAT2 siRNA oligonucleotides as described in the legend to Figure 3. Twenty four h following transfection, we trypsinized 3 wells of the BCAT2 siRNA‐treated cells and combined them into one new well. Similarly, for the control siRNA treated cells, we trypsizined 1 well and simply moved the cells into 1 new well. Cells were allowed to grow in regular GM for another 24 hr. They were then shifted into regular DM and their ability to differentiate was examined. (a) Cell viability was measured in cells transfected with two different BCAT2 siRNA oligonucleotides. Effects of increasing cell number (b) on differentiation (c) in BCAT2‐depeleted cells. For a, data are mean ± *SEM*; *significant difference (*p* < .05) from BCAT2‐siRNA; *n* = 3 independent experiments

Upon BCAT2 transfection, we observed a marked reduction in cell number, especially on D1 and D2 (Figure 3a). By D4 and 5, cell number improved, likely as a result of a diminishing effect of RNAi on BCAT2 level (see Figure 3b). Cell viability was also reduced in BCAT2‐depeleted cells, especially on D2 of differentiation (Figure 4a). We therefore attempted to rescue the differentiation defects by increasing cell number at the time of shift into the DM (Figure 4b; please see Method section). As expected, augmenting cell number increased cell confluency at D0 and D1 of differentiation, as there were minimal empty spaces between cells in the BCAT2 siRNA treatment group (Figure 4b and c compared to Figure 3a). In spite of this, however, BCAT2‐depletedcells still showed an absence of differentiation and exhibited a marked reduction in cell number at D3 of differentiation (Figure 4b), suggesting that the reason BCAT2 deficient myoblasts did not fuse and differentiate was not due to reduced number of adherent cells at the onset of differentiation.

### 3.5 Branched‐chain α‐ketoacid supplementation does not rescue differentiation defects in BCAT2‐depleted myoblasts

**Figure 5:**
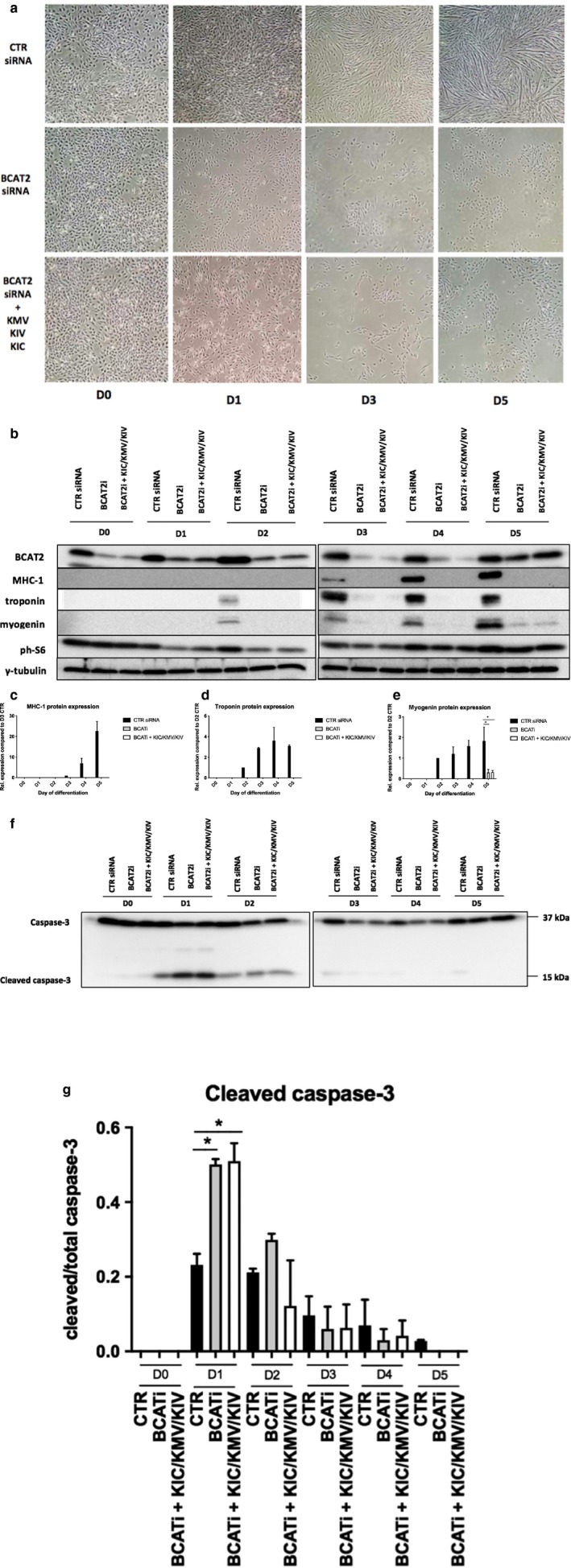
Supplementation with branched‐chain ketoacids does not rescue differentiation defects in cells depleted of BCAT2. Myoblasts transfected with BCAT2 siRNA oligonucleotides as described in the legend to Figure 1 were cultured in a regular DM supplemented with a combination of 200 µM of α‐ketoisocaproate (KIC), 200 µM α‐ketomethylvalerate (KMV), and 200 µM α‐ketoisovalerate (KIV). (a) Light microscope images of cells at time of shifting into DM (D0) and on day 1, (D1), 3 and 5 of differentiation. (b–e) Expression of myogenic proteins MHC‐1, troponin, and myogenin. (f and g) Apoptosis marker caspase 3 expression. Data are mean ± *SEM*; *significant difference (*p* < .05) from BCAT2‐siRNA within same day; *n* = 3 independent experiments. In (f and g), *n* = 2. CTR siRNA: cells transfected with scramble siRNA oligonucleotides; BCAT2i: cells transfected with BCAT2 siRNA oligonucleotides; BCATi + KIC/KMV/KIV: cells transfected with BCAT2 siRNA oligonucleotides and cultured in a medium supplemented with the three α‐ketoacids of the BCAA

Since BCAT2 produces KIC, KMV, and KIV (the ketoacids of leucine, isoleucine, and valine, respectively), we wondered if supplementation of these ketoacids would rescue the differentiation defects seen in BCAT‐2 depleted cells. However, addition of these BCKAs to BCAT2 depleted cells resulted in no visible amelioration of myoblast fusion, cell death, and the expression of myofibrillar proteins, and of myogenin (Figure 5a–e). Hence, the reason BCAT2‐depletion negatively affects myoblast differentiation is likely due to another BCAT2‐mediated function other than BCKA production. Furthermore, supplementation of differentiation medium with vitamin B6 (the co‐enzyme of BCAT2), a‐ketoglutarate, glutamic acid, and glutamine (substrates/products of BCAT2 reaction) did not correct differentiation defects in BCAT2‐depletion defects (data not shown).

### 3.6 BCAT2 depletion induces programmed cell death in myoblasts

To determine the mechanism by which BCAT2 depletion affected cell number, we probed for caspase‐3 protein, which when cleaved, induces programmed cell death via apoptosis (Mukasa, Momoi, & Momoi, 1999). We observed that BCAT2 knockdown significantly increased the amount of cleaved caspase‐3 at D1 of differentiation (*p* < .05) (Figure 5f and g). Supplementing BCKAs to BCAT2‐depleted myoblasts did not rescue levels of cleaved caspase‐3, suggesting that increased apoptosis might be responsible for reduced cell number during differentiation in BCAT2‐depleted cells.

### 3.7 Concurrent knockdown of BDK rescues differentiation defects in BCAT2‐depleted myoblast

**Figure 6:**
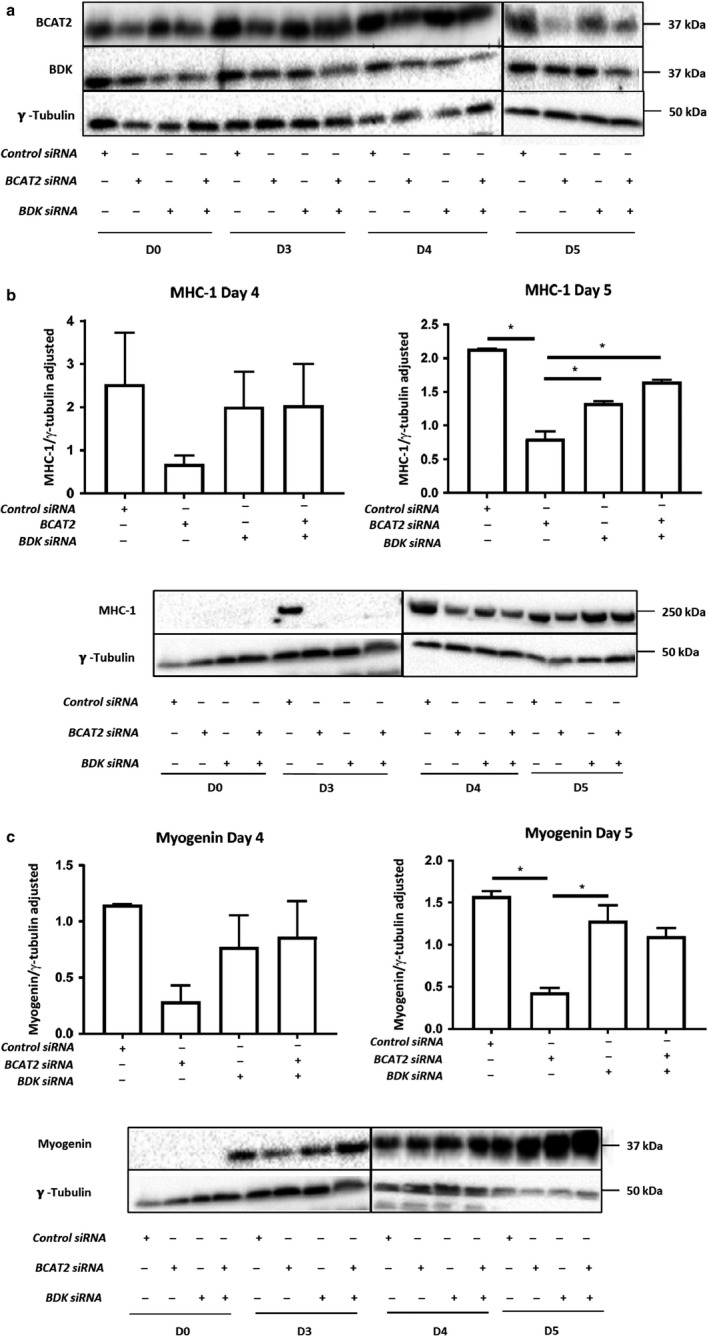
Co‐transfection of BCKD kinase (BDK) siRNA oligonucleotides ameliorates differentiation defects in BCAT2 depleted cells. L6 myoblasts were transfected with BCAT2 siRNA, with or without co‐transfection with BDK siRNA oligonucleotides. Two days later, myoblasts were harvested or shifted into regular DM. Samples were harvested on D3‐D5 of differentiation. (a) Western blot images for BCAT2 and BDK. (b–c) protein abundance of MHC‐1 and myogenin. Data are mean ± *SEM*; *n* = 3 independent experiments; *significant difference in the indicated pair‐wise comparisons (*p* < .05)

Interactions between BCAT2 and BCKD complex promote flux through the BCAA catabolic pathway (Islam et al., 2007). We have shown that knockdown of branched‐chain α‐ketoacid dehydrogenase kinase (BDK), a kinase that phosphorylates and inactivates BCKD, increases BCKD activity and promotes myotube formation (Brendan Beatty and OAJ Adegoke, unpublished observations). Therefore, we examined whether increasing BCKD activity via BDK knockdown (Figure 6a) would rescue differentiation defects observed in BCAT2 knockdown cells. On D4 and 5, concurrent BDK knockdown partially restored MHC and myogenin levels in BCAT2‐depleted cells, and the effect of BDK knockdown was significant on D5 (Figure 6b and c). Thus, concurrent BDK knockdown partially rescues differentiation defects seen in myoblasts with reduced levels of BCAT2.

## 4 DISCUSSION

We have demonstrated a requirement for appropriate regulation of BCAA catabolism for optimal muscle cell differentiation. While a requirement for leucine during differentiation might be expected since leucine is an essential amino acid, we have demonstrated that this requirement is easily met by provision of KIC, the transamination product of leucine. Significantly, even in the presence of sufficient amount of branched‐chain amino acids and their ketoacids, we demonstrated a specific requirement for BCAT2 during the differentiation of muscle cells. The impairment in differentiation in BCAT2‐deficient cells could not be rescued with the provision of the ketoacids of BCAA or with related products of BCAA catabolism but was partially rescued by co‐depletion of BDK, suggesting a role for BCKD in regulating the functions of BCAT2 and therefore muscle cell differentiation.

Evidence to support a role for BCAA in skeletal muscle anabolism, especially with regard to stimulation of protein synthesis and suppression of muscle proteolysis, is incontrovertible. However, skeletal muscle anabolism can occur not only by an increase in protein mass of individual fibers, but also through an increase in myotube number, some of which can fuse with existing myofibers or form new fibers (Yin, Price, & Rudnicki, 2013). Given the myriad of changes that takes place during the differentiation of myoblast to myotubes, for example the formation of myofibrillar proteins, it is remarkable that not much is known about nutritional regulation of this process, more so because nutrients are well known to regulate protein synthesis. Components of the nutrient sensitive pro‐anabolic mTORC1 signaling, including RagA, RagB, and vps34 have all been implicated in myoblast to myotube differentiation (Yoon & Chen, 2013).The fact that leucine requirement for differentiation is met by provision of KIC, the ketoacid of leucine, is consistent with other reports showing that many of the anabolic effects of leucine are duplicated by KIC (Escobar et al., 2010; Lynch et al., 2003). An increase in the abundance of BCAA catabolic enzymes, especially in the protein and mRNA abundance of the catalytic E1α subunit of BCKD during differentiation, and an inability of cells depleted of BCAT2 to differentiate strongly suggest that increased catabolism of these amino acids is vital for differentiation. However, this happens without any apparent change in intracellular BCAA concentrations, suggesting that during differentiation, the transport of these amino acids likely also increases.

Cell‐to‐cell contact is vital for cell fusion and myotube formation (Abmayr & Pavlath, 2012). Although BCAT2 depletion led to decreases in cell number, the fact that impaired differentiation was still observed when cell confluency was increased suggests that decreased cell number on D0 per see was not the cause of impaired differentiation. Rather, in confluent cells, BCAT2 depletion appears to lead to the accumulation of cells that although were in contact with other cells, were simply differentiation incompetent, resulting in increased apoptosis. The mechanisms by which BCAT2 depletion led to decreased cell number or increased apoptosis is not clear. However, BCAT2 mediates decreased cell proliferation observed in pancreatic adenocarcinoma cell lacking mitochondrial malic enzymes 2 and 3 via its supply of glutamine for nucleotide synthesis (Dey et al., 2017). While we cannot rule out a role for BCAT2 in regulating myoblast proliferation, our data suggest a role for the enzyme in regulating apoptosis in postmitotic myocytes.

The fact that provision of the ketoacids of BCAA did not rescue differentiation defects on BCAT2 depleted cells suggest that products other than the BCKA that are linked to BCAT2 might be limiting for myotube formation in cells depleted of BCAT2. These would include α‐ketoglutarate, alanine, glutamic acid (the usual transamination substrate/product of BCAT2 action) and glutamine (from glutamate amidation). BCAT2 knockout mice have up to 80% decline in muscle concentration of alanine, glutamate, and glutamine (She et al., 2010). A lack of myoblast differentiation is likely not due to alanine deficiency as we showed that alanine deprivation did not affect differentiation (Figure 1). Glutamine is metabolized in skeletal muscle and is linked to mTORC1 activation (Jewell et al., 2015). However, the differentiation medium in which the cells were cultured contained 75 mg/L and 292 mg/L of l‐glutamic acid and l‐glutamine, respectively. Further supplementation with glutamine and glutamic acid or addition of α‐ketoglutarate did not rescue differentiation defects. This suggests that other metabolites, for example downstream of the BCKA, might be limiting. It is also possible that BCAT2 interacts with/regulates the functions of other proteins that might be critical for differentiation. Physical interactions between BCAT2 and BCKD promote flux through BCAA catabolic pathway (Islam et al., 2007). A lack of BCAT2 and the subsequent reduction in flow of substrates for downstream metabolism might affect the production of substrates and reduced equivalents (NADH, FADH2) that may regulate myotube formation. This is consistent with our data showing partial correction of differentiation defects in myoblasts with simultaneous knock down of BCAT2 and BDK. These results suggest a role for BCKD in facilitating the functions of BCAT2 during myoblast differentiation.

We showed that myoblasts depleted of BCAT2 have reduced mTORC1 signaling. There is evidence that mTORC1 regulates muscle cell differentiation (Coolican, Samuel, Ewton, McWade, & Florini, 1997; Erbay & Chen, 2001). Because of a need for myofibrillar protein synthesis and the fact that mTORC1 is regulator of protein synthesis, further studies will be needed to clarify whether there is a link between reduced mTORC1 signaling and impaired cell differentiation in myoblasts with reduced BCAT2 levels.

A weakness of this work is that experiments were carried out in an immortalized cell line. Although it would be necessary to confirm whether these observations can be reproduced in primary muscle cells, many of the significant discoveries on the molecular regulation of muscle mass and metabolism were initially identified in vitro models like ours. Therefore, it is likely that our data reflect what is going on in primary muscle cells. Other measures of cell differentiation, including fusion index, could also be used to assess treatment effects on this process. However, changes in the expression of myofibrillar proteins occur in parallel with fusion index and are routinely used to determine treatment effects on differentiation (Millay et al., 2013; Wiles et al., 2015). Finally, our work demonstrating a requirement for leucine during differentiation used leucine‐free medium, which would rarely occur in vivo. It would be interesting to see whether graded levels of leucine have differential effects of myoblast differentiation.

In conclusion, we showed a requirement for BCAT2 during differentiation of muscle cells from myoblasts to myotubes. We demonstrated that impairment in differentiation in BCAT2‐depleted cells could not be corrected by provision of BCAA‐derived α‐ketoacids and other proximal products of the reaction catalyzed by enzyme but that these defects could be partially rescued by co‐depletion of BDK. Because formation of new muscle fibers is needed for muscle repair (Karalaki, Fili, Philippou, & Koutsilieris, 2009), if these data are reproduced in primary myocytes, our findings suggest that interventions that modulate BCAA catabolism might hold promise for treatment of muscle regeneration after damage due to, for example, trauma. Interestingly, impaired BCAA catabolism is seen in insulin resistance (Newgard et al., 2009; Wijekoon, Skinner, Brosnan, & Brosnan, 2004) and related metabolic diseases such as type 2 diabetes (Kuzuya et al., 2008; Lotta et al., 2016) and cardiovascular disease (Uddin et al., 2019), all of which are associated with some degree of muscle wasting (Kim et al., 2014; Park et al., 2009; Tajiri, Kato, Nakayama, & Yamada, 2010; Wang, Hu, Hu, Du, & Mitch, 2006). This raises the possibility that correcting the impairments in BCAA metabolism may be beneficial in treating associated sequelae seen in such diseases.

## CONFLICT OF INTEREST

No conflicts of interest, financial or otherwise, are declared by the authors.

## AUTHOR CONTRIBUTIONS

Z.D. and O.A.J.A. conceived and designed the experiments; Z.D. and G.M. performed experiments and generated figures; Z.D. and O.A.J.A wrote and edited the manuscript; all authors reviewed and approved the final version of the manuscript.
